# Prediction of protein domain boundaries from inverse covariances

**DOI:** 10.1002/prot.24181

**Published:** 2013-02

**Authors:** Michael I Sadowski

**Affiliations:** MRC National Institute for Medical Research, The RidgewayMill Hill, London NW71AA, United Kingdom

**Keywords:** protein structure prediction, protein contact prediction, bioinformatics methods, domain parsing, Kernel density estimation, SCOOBY-DO, FT-COMAR, DomCUT, DOMPRO, DLP-SVM

## Abstract

It has been known even since relatively few structures had been solved that longer protein chains often contain multiple domains, which may fold separately and play the role of reusable functional modules found in many contexts. In many structural biology tasks, in particular structure prediction, it is of great use to be able to identify domains within the structure and analyze these regions separately. However, when using sequence data alone this task has proven exceptionally difficult, with relatively little improvement over the naive method of choosing boundaries based on size distributions of observed domains. The recent significant improvement in contact prediction provides a new source of information for domain prediction. We test several methods for using this information including a kernel smoothing-based approach and methods based on building alpha-carbon models and compare performance with a length-based predictor, a homology search method and four published sequence-based predictors: DOMCUT, DomPRO, DLP-SVM, and SCOOBY-DOmain. We show that the kernel-smoothing method is significantly better than the other *ab initio* predictors when both single-domain and multidomain targets are considered and is not significantly different to the homology-based method. Considering only multidomain targets the kernel-smoothing method outperforms all of the published methods except DLP-SVM. The kernel smoothing method therefore represents a potentially useful improvement to *ab initio* domain prediction. Proteins 2013. © 2012 Wiley Periodicals, Inc.

## INTRODUCTION

The organization of protein structures into discrete structural domains was observed when only a few structures had been solved. For example, the structures of chymotrypsin,^1^ trypsin,^2^ elastase,^3^ papain,^4^ lysozyme,^5^ lactate and malate dehydrogenase,^6^, ^7^ phosphoglycerate kinase,^8^ and thermolysin^9^ all showed multiple “continuous regions,” in the terminology of Wetlaufer's 1973 summary in which the notion of domains was first presented in a unified way.^10^

Since that point there have been many substantial advances in the analysis, delineation, and classification of protein domains using sequence (SMART^11^; PFam^12^) and structure (SCOP^13^; CATH^14^), with important insights into their functional promiscuity and evolution^15^, ^16^ as well as the folding of individual domains and multidomain proteins.^17^

Identification of structural domains from unannotated sequences is a problem of great importance in structural biology: NMR spectroscopy, crystallization, and biophysical analyses of proteins are made significantly more tractable if domains can be identified prior to expression. Computational analyses are also substantially improved after domain identification: iterated homology searches, for example, are considerably less prone to profile drift leading to inclusion of unrelated sequences and a lack of profile information when single domains are used as queries^18^ and many more computationally intensive methods (e.g. *ab initio* structure predictions) have high-order time complexity dependence on the length of the protein chain and are impractical for longer proteins.^19^, ^20^

Given the great interest in this problem there has, naturally, been substantial effort directed toward computational methods for domain identification. Principal sources of information which have been exploited are domain length distributions,^21^ information from sequence similarity searches,^22^ and hydrophobicity taken either from single sequences or sequence profiles.^23^, ^24^ The main conclusion which must be drawn from these studies is that domain prediction is extremely difficult, a conclusion also reached during the earlier CASP competitions, in which domain prediction was assessed as a separate category.^25^, ^26^ Consequently, the state of the art is not significantly advanced from the naive approach. One source of the difficulty is the ambiguity in domain parsing, as identified by comparison of structural domain classifications^27^ and discussed at length by Holland *et al*.^28^

Recently, the application of sparse inverse covariance matrices to contact prediction has led to a significant improvement in the accuracy of such predictions,^29–34^ raising the possibility of using such predictions as a source of information on possible domain boundaries, an earlier study of which was performed by Rigden.^35^ In this article, we explore a number of methods to exploit this new information and show that a simple kernel-smoothing predictor can provide accurate information of use in domain prediction with an improvement of between 8 and 20% over other *ab initio* predictors.

## METHODS

### Predicting domains from contact data

The use of predicted contacts for domain prediction gives us the advantage of being able to apply techniques previously developed for parsing domains from structures by reconstructing rough CA models using distance-geometry methods such as FT-COMAR.^36^ While a substantial number of such approaches have been developed^37–43^ our preliminary tests quickly found that most methods would not accept the rough models produced by FT-COMAR, while others were no longer available. Three approaches which required only alpha-carbon models were therefore tested: **PDP**,^38^ Taylor's **dom** method,^39^ and **domain1.2**,^37^ developed by the Sternberg group. These particular methods were the only three readily available methods which use only α-carbon features to determine domain cuts and which are therefore suitable for use with FT-COMAR models.

Each method has essentially two parameters: the size of contact list used to make a prediction (*N* above) and the contact distance, *D*, a parameter for FT-COMAR. Testing with real contact data demonstrated that high values of *N* were undesirable, as were high values of *D*. The explanation for this is that large numbers of (noisy) contacts produce models which are roughly spherical, leading to predictions of only single domains, while the *D* parameter needs to approximate the characteristic length scale expected by the domain parsers. For all methods values of 2000 for *N* and 8 for *D* were found to perform best (data not shown) and are used in the results presented here. Domain parsing methods were used with default settings. In the case of **dom** and **domain1.2,** which make multiple predictions, only the final prediction was used.

We also developed a faster method based on kernel density estimation (KDE). Following Rigden,^35^ we implemented a method for estimating the likely positions of domain boundaries based on contact density without constructing models. The premiss of the method is that there will be more predicted contacts for residues within the same domain than for residues in different domains, with noise being distributed at random intervals and intradomain contacts generally fewer in number than interdomain contacts. The method therefore assigns the probability of each residue position being within a domain by determining how many predicted contacts would be broken if the chain were to be split at that point. A smoothed PDF of the cut points is derived using Gaussian kernel density estimation^44^, ^45^ as follows: for each predicted contact we iterate over the residues between those in contact, placing a Gaussian with a particular standard deviation (the bandwidth parameter) centered on each residue. The cumulative density across the whole sequence is then summed and normalized to define a smoothed PDF representing the probability of disrupting a (predicted) contact if a domain boundary is predicted at that point. To make a prediction, local minima in the smoothed PDF are found by estimating the first derivative of the curve at each position using a window of 5 residues either side. Each minimum is then defined as a cut-point.

For all domain targets, the highest scoring 1000 predicted contacts (filtered to a minimum sequence distance of 5 residues) were used as inputs to the method. A variety of functions for setting the bandwidth parameter were tested using fixed values and linear, logarithmic, and fractional power functions of the sequence length with parameters as follows: *L/n* for values of *n* = 1, 5, 10, 15, 20, 30 for the linear estimator; log_*k*_ (*L*) for *k* = 1–9 inclusive for the logarithmic function and *L*^1/*x*^ for *x* = 2–9 inclusive. Fixed values of 1, 5, 10, 20, 30, 40, and 50 were tested. We also used the asymptotic mean square error (AMISE) optimal bandwidth calculated using the secant method. The KDE method was implemented in PERL, using the CPAN module Statistics::KernelEstimation–0.05 for kernel density estimation. Source code for all new methods is available for download from: mathbio.nimr.mrc.ac.uk/wiki/Software.

### Domain prediction targets, contact predictions, and structural contact definition

We used data from two sources to test the methods. The 153-protein set defined by Holland *et al*.^28^ for testing domain parsers (we refer to this as “the Bourne set”) and the set of 221 targets from the CASP 7 and 8 experiments. For comparison purposes, we pooled the data into a set of 374 proteins, referred to as the “full dataset.”

Contact predictions were made as follows: the sequence of each chain in the set was used as a query to search Uniref100 using the jackhmmer method from the HMMer 3.0 package^46^ with three iterations, all other parameters default. Contact predictions were made using the ranked results of sparse inverse covariance matrix estimation with the graphical lasso.^30^, ^47^ Our implementation of this predictor was based on the PSICOV method^30^ with the minor difference that ρ parameters were set at 1 for diagonal elements, 0.001 otherwise.^37^ Briefly, the method works as follows: the alignment is used to derive a symmetric *L*× *L* × 21 × 21 matrix (*L* being the length of the target sequence) where each entry 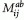 is defined as the covariance between amino acids *i* and *j* in the alignment columns corresponding to positions *a* and *b* in the sequence, gaps being treated as a 21st amino acid character. This has the following equation:

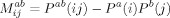


The graphical lasso method^47^ is an efficient way of inverting this matrix, which is very large for long sequences. Finding the inverse has the effect of reducing the covariance signals to only those resulting from direct contacts between amino acids, removing a significant portion of the noise, and substantially improving contact prediction.^30^

Contacts were derived from real structures by finding a pseudo-C_β_ based on the α-carbon coordinates as follows: for three consecutive Cαs C_1_, C_2_,C_3_ find the image B of C_2_ in the line C_1_–C_3_ and define the pseudo-C_β_ as the point 2 Å from C_2_ along the line from B to C_2_.^48^ Inter-residue distances were defined as the distance between these pseudo-C_β_ atoms for all residue types and contacts were defined for distances of ≤ 8 Å.

### Other domain predictors

As comparison measures we implemented two alternative methods of domain prediction which have previously been shown to perform well: a naive predictor using only length information inspired by the DGS method^21^ and a homology search-based method which identified endpoints of alignments to CATH domain HMMs (v. 3.2)^14^ and used a simple smoothing protocol to derive predictions.

The naive predictor was a simple Bayesian method using KDE to determine PDFs for the probability of a chain having a particular length given that it has 1, 2, 3, or 4+ domains. This was then used to find *P*(*N*|*L*), *N* being the number of domains and *L* the chain length. For each value of *N* we found the length-independent probability of a domain boundary at each location in a sequence, all sequences being scaled to length 1000. Fractional parts of the profile were summed when making predictions (e.g. for a protein of length 100, the density for cell 0 would be the sum of cells 0–9 in the profile, cell 1 would be the sum of cells 10–19, etc.). The probability of each residue being a domain boundary was multiplied with the probability of the corresponding value of *N* to produce a final prediction. Cuts were then made wherever peaks were found in the profile using the estimated first derivative calculated as for the KDE method. The method was parameterized using domain length distributions and cut points derived from the CATH database (v3.2).^14^

The homology-based predictor used HMMER3.0 to search the CATH Gene3D HMM library (v 3.2). Endpoints of alignments were then assembled into a profile which was smoothed by repeated averaging. Domain boundaries were predicted based on the positions of local peaks within the endpoint profile, forbidding boundaries closer than 60 amino acids to one another or to the sequence termini. See Supporting Information for further details.

To further assess the performance of the methods, we compared them to four published domain boundary prediction methods: DOMCUT,^49^ DomPRO,^50^ DLP-SVM,^51^ and SCOOBY-Domain.^25^, ^26^ The DOMCUT and DVLP-SVM methods were accessed using the servers provided by the authors at http://www.bork.embl-heidelberg.de/Docu/mikita/domplot.cgi and http://www.tuat.ac.jp/∼domserv/cgi-bin/DLP-SVM.cgi, respectively. DLP-SVM predictions were taken from the SVM-ALL category of server results. DomPRO and SCOOBY-DOmain were run locally using default parameters. Full details of the methods can be found in Supporting Information.

### Assessment of domain predictions and domain prediction methods

Following the assessment of domains in earlier CASP experiments, we used the normalized domain overlap (NDO) score to determine the accuracy of predictions.^25^ The score algorithm worked as follows: for each domain the relevant annotations were used to define domain segments labeled 1, 2, 3, etc., according to the positioning of their first residue. Each residue was labeled with its appropriate state. In the same fashion, predicted domain segments were given ordinal labels based on the location of the first residue of each segment. The NDO score is then simply

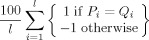

where *l* is the number of labeled residues and *P*_*i*_ (*Q*_*i*_) is the label of the protein (prediction) at residue *i*. Scores were truncated at 0 to produce values in the interval [0–100]. Predicted linker elements were given no label and as such were ignored, thus *l* is equal to the length of the protein minus the number of unlabeled residues.

To avoid over-zealous penalties from label mismatches (i.e. where a short discontinuous segment occurred early in the chain) label matching was optimized using the stable marriage algorithm^52^ with preferences determined by the size of the overlap between each label type. Briefly, this algorithm seeks to find the best pairings for members of two sets given a matrix of preferences, therefore finding the optimal match between predicted domain labels and the annotated correct labeling. Thus in this case if a discontinuous domain is labeled as occupying position 1 and positions 100–200 and the correct answer has no discontinuities the large overlap between predicted domain 1 and actual domain 2 would lead to the labels being swapped. This prevents trivial mistakes from reducing the NDO score artificially since the labeling is arbitrary.

To determine whether the results were statistically robust, we performed all-v-all paired-samples Wilcoxon signed-rank tests. The rationale behind using a nonparametric test is that the underlying data do not fulfill the strict requirements for a *t*-test as the data are not normally distributed. 45 such tests were performed both on the full dataset and on the subset of multidomain proteins to separately assess the ability of the methods to make predictions in a realistic situation and to predict domain boundaries. The resulting *p*-values were corrected for multiple testing using the Bonferroni correction and significant differences were assessed using critical values for a two-tailed test at 5% significance after applying the multiple testing correction.

## RESULTS

### Optimizing bandwidth for KDE

**Figure 1:**
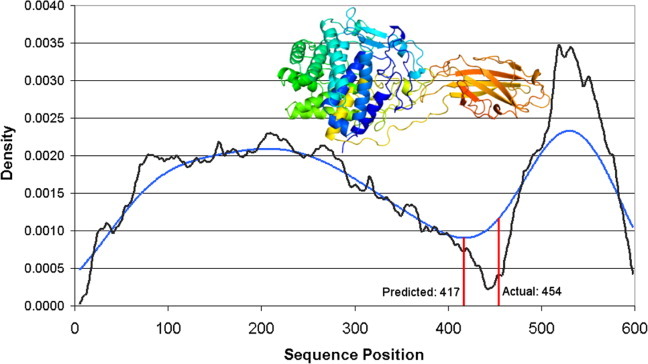
Smoothing contact profiles with kernel density estimation. The predicted contact profile for protein 3TF4 is shown before (black line) and after (blue line) kernel density estimation smoothing with a bandwidth of 40. Red lines show the positions of the real and predicted domain boundaries. A PyMol^53^ ribbon diagram of the structure colored blue–red N–C is shown above the line.

The single parameter for the KDE method is the bandwidth used in the kernel density estimation step. For Gaussian kernels as used here this controls the standard deviation of the kernels used, σ, and therefore produces smoother profiles for larger values of σ. Consequently, low bandwidths produce undersmoothed PDFs which lead in many cases to overprediction while high bandwidths lead to oversmoothed PDFs and underprediction, favoring single-domain cases. Figure 1 depicts the predicted contact profile for a two-domain protein to show the effect of smoothing. Tests were performed using real contact data with pseudo-Cβ atoms and a threshold of 8Å with the Bourne set.

Optimal bandwidths can be estimated from the data by minimizing the estimated asymptotic mean integrated square error (AMISE). The secant method was used for this purpose to provide initial values for testing. Observation of the profiles generated suggested that they were somewhat oversensitive to noisy predictions and generally resulted in overprediction of domain boundaries. We therefore tested the following functions of domain length as bandwidth parameters: fixed bandwidths (1, 5, 10, 20, 30, 40, 50), linear scaling (l/1, l/10, l/15, l/20, l/30, l/40, l/50), power scaling (l^1/2, l^1/3, l^1/4, l^1/5) and logarithmic scaling (bases 2, 3, 4, 5, 6, 7, 8, 9).

Fixed bandwidths led to the expected result that an increase in bandwidth produced fewer domain predictions and systematically favored the single-domain examples in the test set over the multidomain examples. We found a value of 5 represented a reasonable balance for the set in question but this still results in substantial overprediction (data not shown). Overall the best function was linear scaling which essentially defined the Pareto front for the method (the set of parameters for which no other parameters are better on both criteria), although square-root scaling was very close in performance to linear prediction with a length quotient of 15. Figure S1 (Supporting Information) plots the mean NDO scores for single vs. multidomain proteins for each threshold value, with the points on the Pareto front for the method labeled with parameter values. The best mean prediction was found for the l/15 choice. The kernel smoothing parameter was therefore chosen as l/15 for subsequent tests.

### Performance with real contacts

We ran the four contact-based predictors on the Bourne set using the real contact data (see methods) anddetermined the overall prediction accuracy for the set as well as the comparative accuracy of single vs. multidomain proteins using the normalized domain overlap (NDO) measure, following the CASP assessments.^25^

**Figure 2:**
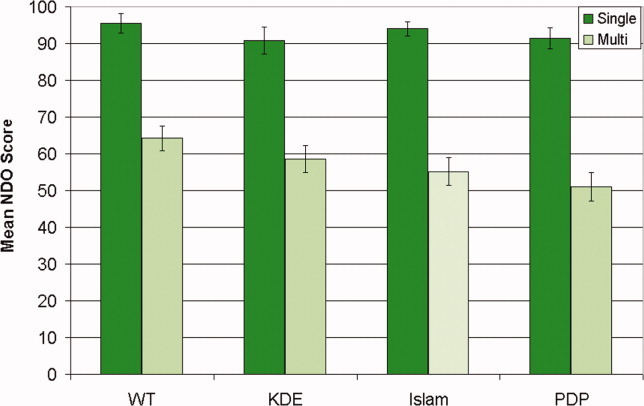
Domain prediction accuracy using real contacts. The three methods using 3D data (Taylor, domain1.2, PDP) and the kernel-smoothing method (KDE) are plotted.

Figure 2 shows the mean NDO scores for the contact-based methods using the top 2000 contacts derived from the structures with the pseudo-Cβ based contact method and setting the FT-COMAR threshold parameter to 12 Å (the top 1000 predicted contacts were used for the KDE method). This is a demonstration of the performance of each of the methods where the contact predictions to be perfectly accurate. On this set the Taylor method is clearly best, with the KDE-based method and Islam methods performing similarly. Examining only the performance on multidomain targets shows that the KDE method is almost identical to the Taylor method, showing that the slight improvement in performance is the result of an improvement on single-domain targets. Removing discontinuous domains (which the KDE method does not predict) improves the performance of the KDE method marginally with respect to the Taylor method but does not significantly change the result (data not shown).

### Performance with predicted contacts

**Figure 3:**
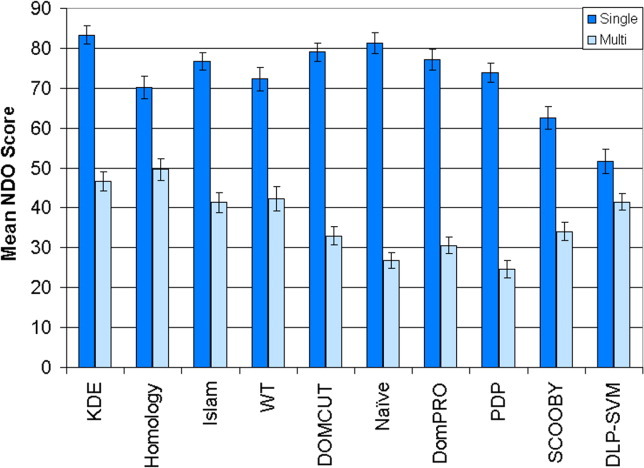
Domain prediction accuracy. The 10 methods are compared based on the mean NDO scores for the single-domain and multidomain targets from the combined CASP and Bourne datasets. Error bars indicate standard errors.

The four contact-based predictors were then run with predicted contact data from our implementation of the PSICOV method,^33^ otherwise in the same way as above, using the full dataset comprising CASP 7/8 targets and the Bourne sequences. For comparison we also ran the SCOOBY-DOmain method, the methods DOMCUT, DomPRO, and DLP-SVM, our homology-based predictor and the naive length-based predictor. Figure 3 shows the performance of the seven methods with predicted contacts rather than those taken from the structures. Taylor's **dom** method performs excellently for multidomain proteins; however, the noise in the predicted contacts leads to overprediction of the single-domain proteins. By contrast the KDE-based method performs extremely well for single-domain proteins with only marginally worse accuracy for multidomain boundaries. Both homology-based predictors are very accurate for multidomain boundaries but are less effective than the KDE method for single-domain proteins. The sequence-based predictor, SCOOBY-DOmain, performs poorly by comparison with these other methods, overpredicting on single-domain proteins with moderate performance for multidomain proteins. The naive predictor is only moderately worse for multidomain proteins while being substantially better for single-domain proteins. **PDP** does not work well with FT-COMAR models, performing worst of all the methods in this instance.

To determine the significance of these differences we performed pairwise Wilcoxon signed-rank tests for paired data between each pair of methods. Tables I and II show the results of this on the full dataset (including both single-domain and multidomain proteins) and the subset of multidomain proteins, respectively. Significant differences were assessed at the 5% level for a two-tailed test after Bonferroni correction of the *P*-values (*n* = 45 tests). Tests which showed significant differences are highlighted in the tables in red and blue.

**Table I:** Statistical Comparisons Between Methods

	KDE	WT	ISLM	PDP	CUT	PRO	DLP	SCO	HOM	DGS
KDE		8.49	6.00	15.0	8.49	10.5	19.8	17.0	0.00	0.00
WT	3e–03		0.00	5.22	0.00	0.00	14.8	9.20	0.00	0.00
ISLM	3e–06	1.00		9.05	0.00	0.00	13.8	11.1	0.00	0.00
PDP	1e–14	2e–08	3e–05		−6.55	−4.51	0.00	0.00	−9.08	−5.12
CUT	0.006	1.00	1.00	9e–05		0.00	11.3	8.50	0.00	0.00
PRO	2e–04	1.00	1.00	2e–03	1.00		9.27	0.00	0.00	0.00
DLP	3e–12	9e–06	3e–04	1.00	4e–04	0.04		0.00	−13.8	−9.94
SCO	2e–11	7e–05	0.03	1.00	4e–03	0.20	1.00		−11.2	−7.17
HOM	0.72	1.00	1.00	4e–07	1.00	1.00	1e–07	2e–07		0.00
DGS	1.00	1.00	1.00	1e–07	1.00	1.00	0.01	2e–04	1.00	

NDO scores for all 368 targets were compared using paired Wilcoxon signed-rank tests. Entries below the diagonal show Bonferroni-corrected *P*-values for the test (*N* = 45 tests). Entries above the diagonal show the mean differences between the two groups, row – column. Cells representing significantly different methods (5% threshold) are colored red if the mean difference is positive, blue if negative. Key to methods: KDE: kernel smoothing, WT: DOM-parsing of preliminary structures, ISLM: Domain1.2 parsing of preliminary structures, PDP: PDP parsing of preliminary structures, CUT: DOMCUT, PRO: DomPRO, SCO: SCOOBY-DOmain, HOM: homology method, DGS: naive length-based predictor.

**Table II:** Statistical Comparisons Between Methods

	KDE	WT	ISLM	PDP	CUT	PRO	DLP	SCO	HOM	DGS
KDE		0.00	0.00	22.1	13.7	16.0	0.00	12.4	0.00	19.7
WT	1.00		0.00	18.5	0.00	0.00	0.00	0.00	0.00	14.8
ISLM	1.00	1.00		16.8	0.00	10.7	0.00	0.00	0.00	14.7
PDP	5e–12	7e–07	3e–06		0.00	0.00	−16.9	0.00	−25.1	0.00
CUT	3e–03	1.00	0.540	0.07		0.00	0.00	0.00	−16.7	0.00
PRO	3e–06	0.09	8e–03	0.87	1.00		−10.9	0.00	−19.0	0.00
DLP	1.00	1.00	1.00	3e–06	0.14	0.03		0.00	0.00	14.8
SCO	0.03	1.00	1.00	0.06	1.00	1.00	0.12		−15.9	0.00
HOM	1.00	1.00	0.73	2e–11	2e–06	1e–04	0.43	2e–05		23.2
DGS	3e–07	4e–03	3e–04	1.00	1.00	1.00	2e–04	1.00	2e–09	

NDO scores for the 165 multidomain targets were compared using paired Wilcoxon signed-rank tests. Entries below the diagonal show Bonferroni-corrected *P*-values for the test (*N* = 45 tests). Entries above the diagonal show the mean differences between the two groups, row – column. Cells representing significantly different methods (5% threshold) are colored red if the mean difference is positive, blue if negative. Key to methods: KDE: kernel smoothing, WT: DOM-parsing of preliminary structures, ISLM: Domain1.2 parsing of preliminary structures, PDP: PDP parsing of preliminary structures, CUT: DOMCUT, PRO: DomPRO, SCO: SCOOBY-DOmain, HOM: homology method, DGS: naive length-based predictor.

From these results, it can be seen that the KDE method is significantly better than all of the other methods except the naive and homology-based predictors when the full dataset is considered. The mean differences range between 6 and 19.8 increases in NDO scores across all methods, 8.49 and 19.8 when compared to the other four published predictors. Using PDP to parse domains is inferior to seven of the nine other methods, suggesting that it is not an appropriate method to use with FT-COMAR's rough models.

Although it is important not to overpredict domain boundaries, requiring the incorporation of single-domain proteins in the dataset, by assessing results using all data we are implicitly assuming a given distribution of single-domain vs. multidomain proteins. The actual distribution might depend on the context of the predictions – Eukaryotes and Prokaryotes, for example, tend to have different distributions of multidomain proteins. Therefore, it is important to assess the quality of predictions on multidomain proteins only, effectively assessing the probability of correctly predicting domain boundaries given that the protein is multidomain.

Table II shows that when we consider only multidomain proteins a slightly different picture emerges: the KDE method is significantly better than the DOMCUT, DomPRO, and SCOOBY methods but no longer provides a significant improvement over the DLP-SVM method. Differences in performance between KDE, DOM, and Domain1.2 are also no longer significant while there is a very large difference between the better methods (KDE, Taylor, Islam, Homology) and the naive DGS predictor which uses only length information. The ability of this method to make good predictions across the whole dataset is therefore attributable almost entirely to its tendency to predict single-domain proteins accurately. DLP-SVM is shown here to be reasonably accurate given only multidomain proteins but tends to substantially overpredict.

Since FT-COMAR can generate multiple models from a single input we tested two methods for deriving a consensus prediction using an ensemble of models to determine whether this could improve domain prediction. We found that re-estimating contacts from an ensemble of models produced a promising increase in contact prediction performance (Supporting Information Fig. S2) but that this did not generally improve domain prediction accuracy (Supporting Information).

## DISCUSSION

Prediction of domain boundaries from sequence remains extremely challenging. Where a known structure for a domain exists and can be aligned to the query sequence predictions can often be quite accurate, as demonstrated by our results and those of others. However, this relies on both the existence and the detection of the known structure and where this is not possible such methods will fail.

We have shown that using the new, more accurate contact predictions derived from inverse covariance analysis can produce domain boundary predictions which are equivalent to or slightly better than those produced by the template-based method and represent a substantial improvement over the four *ab initio* predictors tested here, providing similar performance to the use of homologous templates. This therefore represents an improvement to the state of the art in *ab initio* domain prediction which will be useful in supplementing the template-based approach.

There remain two areas in which the kernel smoothing method could be improved: first it takes no account of discontinuous domains, which often result in inaccurate predictions. Using the structural domain parsers, which already account for this feature of domains, is one way in which this could be mitigated although our analysis suggests that in fact the level of accuracy on discontinuous domains is similar (data not shown). Regardless of this an improved model which accounts for this might prove very useful in improving accuracy. Second the method is not always successful for domains which are not fully compact, e.g. barrel structures, which are frequently split in two. Although from an evolutionary point of view the existence of half-barrels might suggest that this is not entirely an inaccurate prediction from the point of view of predicting structure it is undesirable to split barrels up. Improvements to the model could also be made to account for this.

Finally the gap between the performance of all methods with real and predicted contacts strongly suggests that further improvements to the contact prediction method would be an important source of increased accuracy in domain prediction.
