# Supplementary material for: Prediction of protein domain boundaries from inverse covariances
Source: Proteins. 2013 Feb;81(2):253–60. doi: 10.1002/prot.24181 (PMC3563215; doi:10.1002/prot.24181)
Supplement: Supplementary file 1 [file prot0081-0253-sd1.pdf]

## Supplementary Information

## Method Descriptions

### *KDE Method*

The kernel-smoothing method takes as input a sorted list of contacts, filtered to a minimum sequence distance of 5. The exact sorting function depends on the source of the data: contacts derived from structure are sorted closest-first while predicted contacts are sorted in order of prediction score, highest first. For reasons of speed only the top 1000 such contacts are typically examined (preliminary testing revealed that the method was not very sensitive to this parameter).

The method uses the contact data to estimate the relative density of contacts which would be broken if a domain cut is predicted to occur at each residue. Thus we maintain a counter for each residue in the sequence and for each predicted contact all residues in the closed interval with the predicted contacting residues as the endpoints have their counters incremented. Local minima in this profile are then likely to indicate domain boundaries assuming that the domains are similar in size and contact themselves more than they contact each other.

The raw profile produced from this is very noisy, (see figure 1 in the main text) and it can be difficult in general to distinguish genuine minima from spurious minima. Smoothing using kernel density estimation (KDE) is a principled, widely-used way to deal with this problem. Intuitively the idea is that the information for a count at a particular residue is not restricted only to that residue but also its neighbours. Technically this is done by changing the discrete counts to the sum of Gaussian distributions centred on each position. The standard deviation of each Gaussian (which is known as the bandwidth

parameter in KDE) determines how far along the chain the information is propagated: large bandwidth leads to profiles which are nearly flat while small bandwidth leads to profiles barely less noisy than those in the original discrete case.

Finally the smoothed profile can be used to estimate cut points by looking for local minima in the profile. In this method the value of the derivative of the curve is estimated at each point k as

$$\frac{d\Phi}{dk} = \frac{\Phi(k+5) - \Phi(k-5)}{11}$$

Local minima are then identified as points where the derivative changes sign from negative to positive. All such minima are predicted as cut-points.

*Model Reconstruction Methods*

The three methods based on previously published domain parsers (WT, Islam, PDP) are identical except for the specific domain parsers used. The input in each case is a list of predicted contacts sorted best-first as described above. Each method then has two parameters: N, the number of predicted contacts to use in model reconstruction and D, the distance threshold which FT-COMAR optimizes each pair of contacts to. Both of these parameters apply only to the domain reconstruction; although the domain parsers do in some cases have their own parameters these were not varied in the experiments.

Predicted contacts are converted to the matrix format required by FT-COMAR and then this method is run with the resulting matrix and the D parameter specified. Neighbour filtering is not used. The resulting model is then parsed for domains with the appropriate domain parsing method.

Where an ensemble prediction was made the FT-COMAR method was run 100 times) and predictions were made for all models. The NDO between each pair of predictions was assessed and the prediction with the highest mean NDO against all other predictions was taken as the ensemble prediction. This method was tested on the WT method only and found not to significantly improve predictions.

*Homology based method*

The homology-based predictor used HMMER3.0 to search the CATH Gene3D HMM library (v 3.2). A profile of endpoints of alignments to these domains was constructed by summing counts at each sequence location, following which the profile was smoothed by taking average values over 7-residue windows across the whole sequence. This smoothing procedure was repeated five times to remove artefacts created by spurious hits. Using statistics taken from CATH (3.2) we found the mean single-domain length to be 130 with a standard deviation of roughly 70. Sequences shorter than the mean length were automatically predicted to be single domain. Cut points were then edited to remove those <50 residues away from either terminus.

*Domain Parser Comparison*

Although it is not central to the questions of this study we were interested to determine whether the three parsers tested performed similarly when given the real structures. This is useful because it can provide some notion of whether good performance on real structures is related to good performance on the crude models. The three methods were run on the structures in the Bourne set and the NDO scores for all targets and

multidomain targets only were compared using Wilcoxon signed-rank tests for paired data as described in the main Methods section. Raw data are shown in table S1. None of the methods were found to be significantly different in their domain parsing performance.

| Method | Mean Single (SD) | Mean Multi (SD) | Mean all (SD) |
|---|---|---|---|
| WT | 81.5 (35.4) | 69.7 (30.6) | 73.6 (32.6) |
| Islam | 88.1 (24.4) | 62.9 (33.7) | 71.4 (33.0) |
| PDP | 80.2 (32.9) | 61.8 (35.6) | 67.9 (35.7) |

Table S1 – Structural domain parsing performance for the Bourne dataset

*Parameter Estimation*

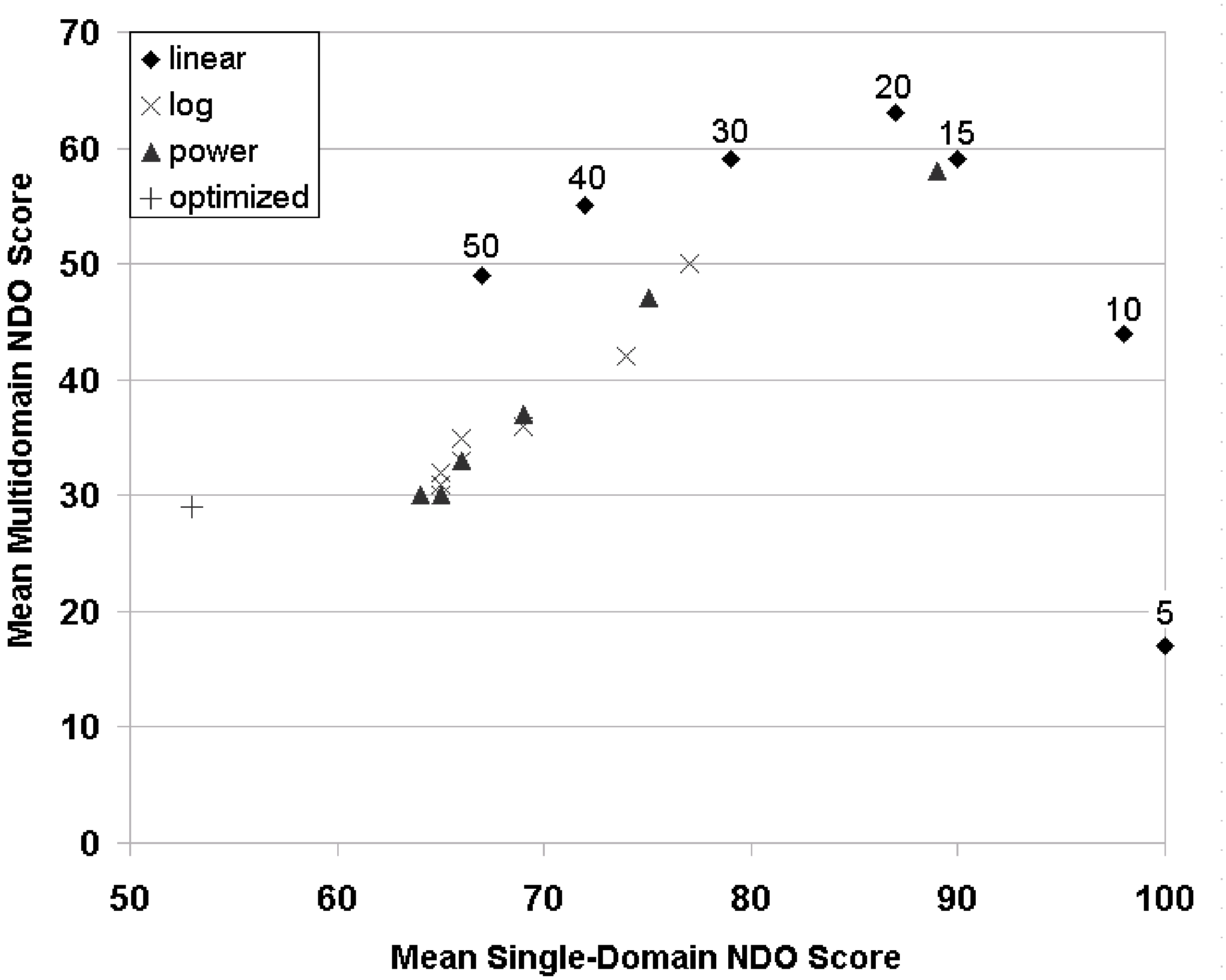


**Figure S1**: Parameter testing using real contact data. The kernel smoothing parameter was tested using optimal AMISE smoothing and a variety of functions of the input sequence length (see Methods). For each function and parameter, the mean single-domain NDO score (x-axis) is plotted against the mean multi-domain NDO score (y-axis), calculated over the Bourne set with real contacts as inputs . Points along the Pareto-front are labelled with parameter values.

*Consensus Predictions and Distances*

Since FT-COMAR uses a stochastic method the model generated in a particular run will differ to some extent (sometimes radically) from models generated on other runs. We investigated using this information to improve the structural domain parser results by generating an ensemble of 100 structures and finding the consensus prediction over the models, the similarity between predictions being assessed using the normalized domain overlap (see below). Additionally we re-estimated contacts by finding the proportion of models in which each residue pair was found below a threshold distance. We then used the closest N of these as predicted contacts for input into the four contact-based predictors and determined whether these produced better results for the KDE and structural methods.

Constructing models from predicted contacts by distance geometry can help to resolve inconsistencies resulting from noise in the predictions. We therefore expected that the mean distances found in the ensemble of models could improve the accuracy of the predicted contacts and potentially improve domain predictions.

Figure S2 compares ROC AUC values for the contact predictions compared to real contacts for the original prediction (x axis) and after re-estimation from the ensemble models for a subset of 35 proteins. The figure shows data for reconstructions using 8 , 20 and 30 Angstrom thresholds for the distance parameter to the consensus contact estimator (see Methods) with the top 2000 contacts from the original contact predictions used in the reconstruction at a threshold of 8 Angstroms. For the KDE method the number of predictions used was increased to 3000 since this process inevitably makes the contact

predictions less sparse.

For low thresholds the AUC values improve overall but in many cases the predictions are made worse. Increasing the threshold to 20Å mitigates this problem without reducing the improvement for other cases substantially. Once the threshold reaches 30Å the AUCs are reduced for the majority of targets.

There are likely to be two reasons that we see this effect: firstly the resolution of inconsistencies as described above and secondly the reintroduction of some of the proximities which would appear as “chains” of contacts, the removal of which is the motivation for the DI method. To test the latter we determined the effect of changing the minimum sequence separation in the AUC calculation from 5 to 10 and observed that we obtained the same result, suggesting that there is some real improvement.

We then assessed whether the improvement was transferred to the accuracy of the domain predictions for the contact-based methods but found that the effect on most methods was to significantly decrease prediction accuracy for both single and multidomain targets. The KDE method did not significantly change although mean accuracy on multidomain targets was marginally higher.

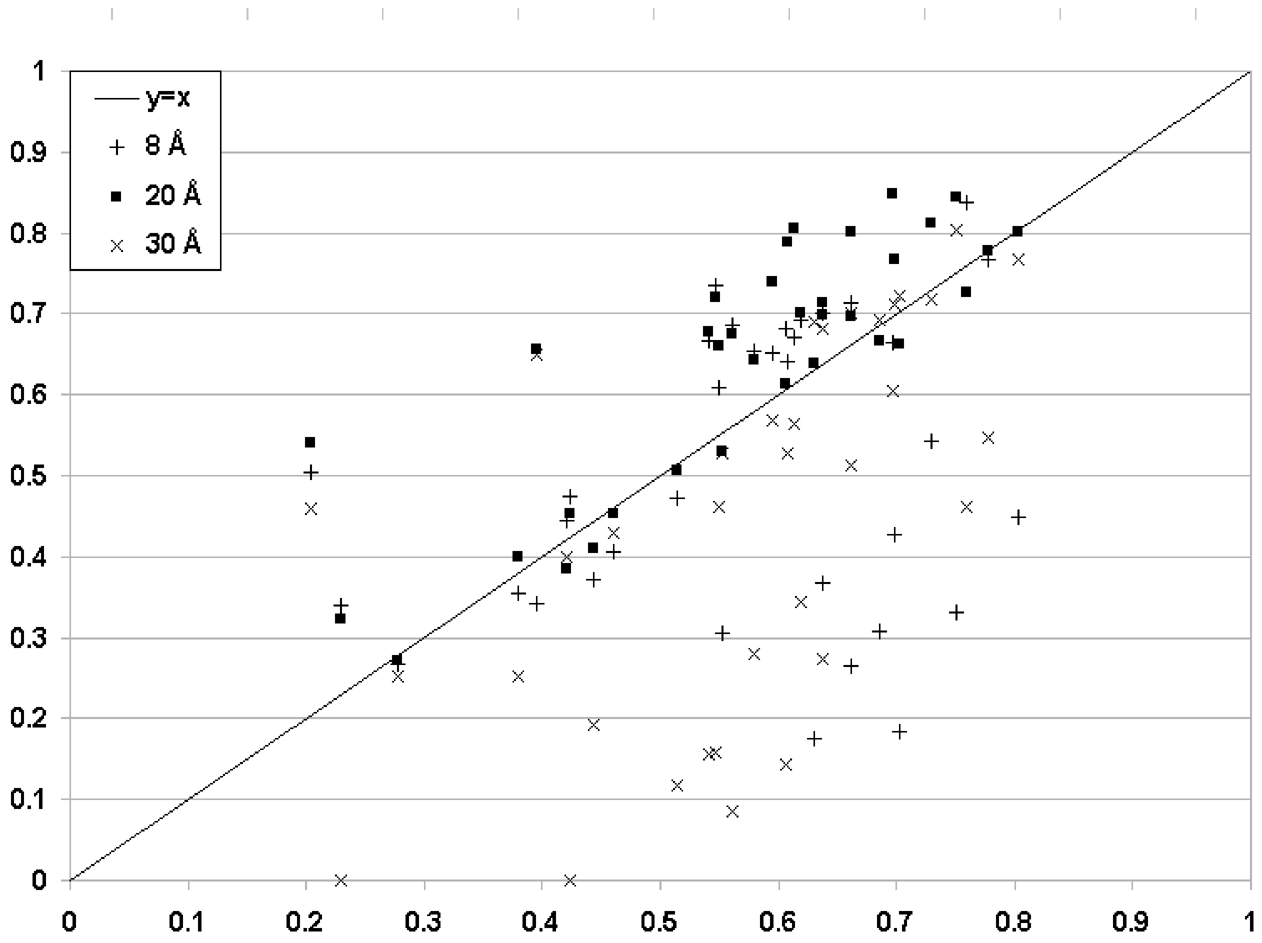


**Figure S2**: Contact prediction improvement by ensemble re-estimation: receiver operator characteristic (ROC) scores for contact predictions are plotted for raw predictions (x-axis) and after re-estimation from 100 FT-COMAR models (y-axis). The y = x line is plotted to assist interpretation. Data are shown for the Bourne set only.
